# Hemophagocytic lymphohistiocytosis, a rare condition in renal transplant - a case report

**DOI:** 10.1590/2175-8239-JBN-2018-0012

**Published:** 2018-10-11

**Authors:** Valentine de A. C. de Castro Lima, Ana Luisa Figueira Gouvêa, Paulo Menezes, Jacqueline da F. Santos, Mayra Carrijo Rochael, Fabiana Rabe Carvalho, Jorge Reis Almeida, Jocemir Ronaldo Lugon

**Affiliations:** 1 Universidade Federal Fluminense, Niterói, RJ, Brasil.

**Keywords:** Kidney Transplantation, Lymphohistiocytosis, Hemophagocytic, Infection, Transplante Renal, Linfohistiocitose, Hemofagocítico, Infecção

## Abstract

Hemophagocytic lymphohistiocytosis (HLH) is an uncommon and life-threating condition characterized by major immune activation and massive cytokine production by mononuclear inflammatory cells, due to defects in cytotoxic lymphocyte function. It is even more unusual in renal transplant recipients, in which it is often associated with uncontrolled infection. The mortality is high in HLH and differential diagnosis with sepsis is a challenge. The approach and management depend on the underlying trigger and comorbidities. We report a case of a 50-year-old renal transplant female admitted with fever and malaise 3 months post-transplant and presenting anemia, fever, hypertriglyceridemia, high levels of serum ferritin, and positive CMV antigenemia. Urine was positive for decoy cells and BKV-DNA. Graft biopsy showed CMV nephritis. Both blood and urine cultures where positive for *E. coli.* Hemophagocytosis was confirmed by bone marrow aspiration. Immunosuppression was reduced, and the patient received high-dose intravenous immunoglobulin and dexamethasone, with complete response after 3 weeks. We highlight the importance of early diagnosis and proper management of a rare and serious condition in a renal transplant patient, which can allow a favorable clinical course and improve survival rate.

## Resumo

A linfohistiocitose hemofagocítica (LHH) é uma condição incomum e potencialmente fatal, caracterizada por importante ativação imunológica e produção maciça de citocinas por células mononucleares inflamatórias, devido a defeitos na função linfocitária citotóxica. É ainda mais incomum em receptores de transplante renal, nos quais está freqüentemente associada a infecções não controladas. A mortalidade da LHH é alta, e o diagnóstico diferencial com sepse é um desafio. A abordagem e o tratamento dependem do gatilho e das comorbidades subjacentes. Relatamos o caso de uma paciente transplantada renal com 50 anos de idade, admitida com febre e mal-estar 3 meses após o transplante, apresentando anemia, febre, hipertrigliceridemia, níveis elevados de ferritina sérica e antigenemia positiva para CMV. A urina mostrou positividade para células decoy e BKV-DNA. A biopsia do enxerto mostrou nefrite por CMV. Ambas as culturas de sangue e urina foram positivas para *E. coli.* A hemofagocitose foi confirmada pelo aspirado de medula óssea. A imunossupressão foi reduzida e a paciente recebeu altas doses de imunoglobulina intravenosa e dexametasona, com resposta completa após 3 semanas. Destaca-se a importância do diagnóstico precoce e do manejo adequado de uma condição rara e grave em um paciente transplantado renal, o que pode permitir um curso clínico favorável e melhorar a taxa de sobrevida.

## INTRODUCTION

Hemophagocytic lymphohistiocytosis (HLH) consists of an immune hyperactivation syndrome that takes place when NK cells and cytotoxic T lymphocytes fail to eliminate activated macrophages, leading to over production of proinflammatory cytokines^1^.

There are primary and acquired causes. Primary HLH is rare and typically manifests in childhood because of two autosomal recessive defects in genes that encode proteins involved in the exocytosis of cytotoxic granules during apoptosis in natural killer (NK) cells.^1^^,^ 2Acquired (secondary) HLH is triggered by various conditions as infections, immunodeficiency, rheumatologic diseases, and cancer.^1^^,^ 3 ^,^ 4 Infection is the most common precipitating factor of HLH in adults, mainly viruses of herpes family (EBV, CMV, HSV, HHV8), but bacterial, fungal, and parasitic pathogens can also be a trigger.^5^^,^ 6

Multiorgan involvement and organomegaly are frequently found and hemophagocytosis results in pancytopenia. Diagnosis of HLH is based on the presence of at least 5 of the following 8 criteria: fever, splenomegaly, cytopenias (affecting ≥ 2 lineages of peripheral blood cells), hypertriglyceridemia and/or hypofibrinogenemia, serum ferritin >500 ng/mL, low activity of NK cells, soluble CD25 > 2.400 U/mL and hemophagocytosis in bone marrow, spleen, or lymph nodes.^7^ Hemophagocytosis is characterized by the presence of red cells, platelets, or white cells in macrophage cytoplasm visualized in bone marrow aspirate or biopsy.^2^

The syndrome is defined by a complex picture. However, some patients may have incipient or partial disease.^1^ Most diagnostic criteria are validated for pediatric patients turning the diagnosis even more difficult in adults.^5^ In fact, clinical features can differ in both groups; children present more commonly hepatomegaly, splenomegaly and jaundice, while adults present serous cavity effusion more frequently.^8^ Therefore, translation of the current HLH guidelines and protocols for the adult population is questionable.^5^

Renal transplant patients are potentially prone to develop HLH due to their immunosuppressive state. Despite that, HLH affects only 0.4 - 2.0% of these patients.^6^^,^ 9 Diagnosis is urgent, since the prognosis of HLH is dismal, with the mortality rate reaching 53% in kidney transplant patients^2^ in comparison with 41% in the general adult population.^2^

Treatment consists of controlling the cause of the HLH and supportive intensive care.^6^ However, this may not be sufficient, and the patient can require specific HLH-treatment, which is indicated when HLH is severe, persistent/recurrent, familial, or genetically verified.^5^ The HLH-treatment is based on etoposide, dexamethasone, cyclosporin A, or hematopoietic cell transplantation, required to prevent recurrence of disease.^1^^,^ 7 Treatment studies in adults have been few and uncontrolled, and the treatment decisions are based on clinical experience.^6^ HLH specific therapy in adults under immunosuppression may include plasma exchange and interleukine-1-directed therapy.^5^ There are some data regarding the use of immunoglobulins as an adjuvant treatment of viral infections associated with HLH.^1^^,^ 2

HLH is an uncommon and serious disease, rare in kidney transplant patients. Triggering factors and other comorbidities can contribute to clinical gravity and mask the signs and symptoms of HLH, making this diagnosis a challenge.^9^

### Case Presentation

A 50-year-old female, undergoing regular hemodialysis for 3 years due to polycystic kidney disease, was submitted to a renal transplant in 2014. By that time, the patient had negative serological tests for CMV, hepatitis B and C, toxoplasma, HIV and syphilis. The donor was a 65-year-old female, CMV-positive, who suffered an ischemic stroke.

The recipient received thymoglobulin as induction therapy and was maintained on prednisone, mycophenolate sodium, and tacrolimus. She underwent universal prophylaxis for CMV infection with intravenous ganciclovir (5 mg/kg) 5 days after transplant, according to the institutional protocol:^10^ twice a day (week 1 and week 2 post-transplant, PT); three times a week (week 3 and week 4 PT); twice a week (week 5 up to week 8 PT), once a week (week 9 up to week 12 PT). The dose of ganciclovir was adjusted for the patient's renal function.

Monitoring of viral reactivation was implemented during the period of use of pharmacological prophylaxis. The patient underwent CMV monitoring by pp65 antigenemia test weekly during first 3 months PT. A PCR test was also performed in plasma at week 8 PT to test the accuracy of pp65 test, as a part of a protocol. The BKV monitoring included urinary tests for decoy cells and RT-PCR biweekly during the first three months PT. RT-PCR for BKV was performed in plasma at end of week 8 and week 12 PT.

**Figure 1:**
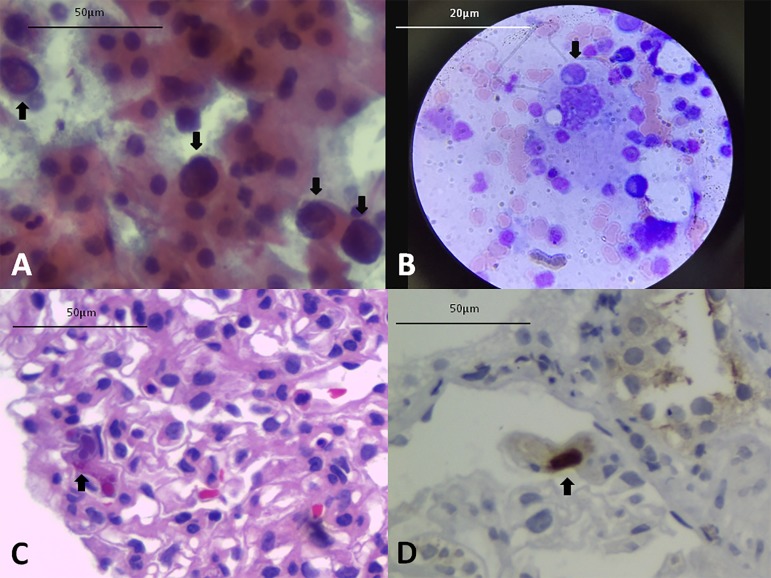
A) Urinary decoy cells (Papanicolaou). B) Hemophagocytosis in bone marrow aspirate (Giemsa). C) Viral cytopathic effect in CMV glomerulitis (Hematoxylin-Eosin). D) Nuclear positive staining for CMV in glomerulus (Immunohistochemistry).

At week 4 PT, a low BK viral load in urine was detected (104.5 copies/mL). Urinary decoy cells were found at week 6 PT (Fig.1A), persisting until week 11 PT. The patient presented positive pp65 test at week 7 (194 cells/200,000 white cells), when the transplant team decided to increase the dosing of ganciclovir. CMV pp65 test persisted positive at week 8 PT (965 cells/200,000 white cells), when CMV DNA was also detected in blood (1294 copies/mL) and clinically significant BK viral load (>10⁷ copies/mL) was detected in urine. BKV was negative in plasma.

At week 10 PT, the patient returned for evaluation with fever, myalgia, and malaise. At week 11 PT on clinical examination, she complained of adynamia, and asthenia, and presented with pallor, tachypnea, and tachycardia. Laboratory analysis showed positive pp65 test (1449 cells/200,000 white cells) anemia (red cells 2.24 x 106/mm3 and hemoglobin 6.7 g/dL), leukocytosis (11,800 x 103/mm3), hypertriglyceridemia (1,500 mg/dL), hyperuricemia (14 mg/dL), and high levels of serum ferritin (7,193 ng/dL). CT scan revealed pleural and pericardial effusions. Blood and urine samples were collected for cultures. Since the patient completed 4 of the 8 HLH-2004 diagnostic criteria^7^, we asked for a hematology consultation. She was then submitted to a bone marrow aspiration, which revealed hemophagocytosis (Fig. 1B). The bone marrow was negative for CMV (immunohistochemistry) and no morphological signs of parvovirus infection were detected. Once HLH diagnosis was confirmed, she received a high dose of intravenous immunoglobulin (400 mg/kg/daily for 4 days) and dexamethasone (weeks 1 and 2 - 10 mg/m 2 daily; weeks 3 and 4 - 5 mg/m 2 daily; weeks 5 and 6 - 2.5 mg/m 2 daily; week 7 - 1.25 mg/m 2 daily; and week 8 - dose tapering to zero).

Both urine and blood cultures were positive for *E. coli*; cefepime was prescribed and immunosuppressive drugs were temporarily withdrawn. A graft biopsy was indicated due to increase of creatinine from 2.3 to 3.9 mg/dL. The sample showed a mild interstitial nephritis and rare nuclear and cytoplasmic inclusions in glomeruli (Fig. 1C) CMV-positive by immunohistochemistry (Fig. 1D). There were no signs of rejection or BKV nephropathy (immunohistochemical staining for SV40 T antigen in the renal tissue and BKV in plasma were negative), despite clinically significant BK viral load (>107 copies/mL) in urine at week 8 and week 12 PT. The patient completed treatment for bacterial infection. She was discharged 20 days after admission in use of prednisone, ganciclovir, tacrolimus, and sirolimus. Laboratory exams at the day of discharge showed normal levels of triglycerides, leukocytes, and serum ferritin of 2,000 ng/dL. The patient also recovered graft function, leaving the hospital with a creatinine level of 2.0 mg/dL. Ganciclovir was maintained until the 4th month PT, after two consecutive negative pp65 tests, one week apart.

## DISCUSSION

HLH is a hyperinflammatory syndrome characterized by overactivation of lymphocytes and macrophages in association with high levels of cytokines.^1^^,^ 4Acquired HLH manifests predominantly in adulthood, most cases presenting first with systemic involvement.^6^^,^ 11

HLH is uncommon in kidney transplant patients, with less than a hundred cases described in international literature^2^^,^ 5 ^,^ 12, despite their immunosuppressive condition and predisposition to infection.^9^Most HLH cases in renal transplant are triggered by reactivation of latent infectious agents precipitated by the immunosuppressive agents.^1^^,^ 2 The diagnosis is urgent, since the prognosis of HLH is dismal, with mortality occurring in 30-50% of patients without therapy. In post-transplant patients, the mortality rate can reach 53%.^2^

The differential diagnosis of HLH with sepsis may be particularly difficult since both conditions may share some clinical and laboratory findings.^1^^,^ 3 ^,^ 11Accordingly, underdiagnose could in part explain the small number of cases reported in this population.^9^It should be mentioned that the HLH-2004 diagnostic criteria^7^were developed for pediatric patients. Identification of HLH in adults may be more difficult and a specific protocol including other variables obtained in laboratory analyses or physical examination may improve HLH diagnosis in this setting.^13^HLH must be considered in patients with prolonged fever of unknown origin and cytopenias.^11^However, sepsis is a cause of hyperinflammation much more common than HLH. Fever, leucopenia, elevated ferritin, hypofibrinogenemia, thrombocytopenia, and deteriorating clinical condition can be present in both sepsis and HLH.^3^There is no single marker to differentiate HLH from sepsis. However, values extremely high of ferritinemia, profound cytopenias, and elevated triglyceride levels (in adults) favor HLH. This patient presented a very high level of ferritin and triglycerides. She presented cytopenia of only one cell lineage (red cells). Although low levels of hemoglobin (<90 g/L) in HLH are more frequent in children than in adults, cytopenia of multiple lineages is less common in adults than in children.^8^However, in transplanted patients, the differential diagnosis can be even more challenging because immunosuppressive drugs may cause pancytopenia, and hypertriglyceridemia may be already present in these patients.^9^She also presented pleural and pericardial effusions, more frequently seen in adult HLH patients.^8^

In fact, treatment of sepsis and HLH include some procedures beneficial for both conditions. Despite that, it is very important to distinguish the two conditions to define the precise treatment, especially in those cases in which the use of cytotoxic drugs are required.^3^^,^ 5

HLH therapy is also based on pediatric data and no specific regimen is available for adult patients in cases of refractory HLH.^5^^,^ 13

Management of HLH in renal transplant patients includes supportive care, immunosuppressive dose reduction, use of specific infectious treatment and high-dose polyvalent immunoglobulin. Although the strategy might be successful in most cases, the best choice in this setting is still a matter of controversy.^9^^,^ 14

In the present case, the patient had coinfections with CMV, BKV, and *E. coli* that were promptly detected. Graft biopsy showed CMV nephritis but not BKV-associated nephropathy. There was no BKV viremia, despite decoy cells shedding and high BK viral load in urine, findings that can be explained by BKV reactivation restricted to the lower urinary tract.

The use of dexamethasone, cefepime, ganciclovir, high dose intravenous immunoglobulin, and decreasing immunosuppressive agents successfully controlled the infections. As a secondary form of HLH, the patient responded to medication, and specific HLH treatment, including cytotoxic drugs, was not needed. Primary HLH should be considered in refractory cases, although it is less common in adults. Familial or inherited forms of the disease can recur, and prolonged therapy and/ or hematopoietic stem cell transplantation (HSCT) may be needed if the patient survives the first episode of disease.

This case report shows the importance of considering HLH diagnosis in transplant population, especially during the first 6 months after transplant, a period in which the immunosuppressive state is more intense and the susceptibility to infections is high. Monitoring for reactivation of viral infections is a valuable tool to early definition of the etiology of the syndrome in infectious secondary forms of disease, allowing a target-oriented therapy. Although a therapy with less toxicity can be effective in adult patients, we should be aware that refractory cases might demand early specific therapy that can be lifesaving.
